# Milk IgA promotes symbionts and limits pathobionts in the early life gut

**DOI:** 10.1093/ismejo/wraf266

**Published:** 2025-12-01

**Authors:** Katherine Donald, Antonio Serapio-Palacios, Tahereh Bozorgmehr, Mahebali Tabusi, B Brett Finlay

**Affiliations:** Department of Microbiology & Immunology, University of British Columbia, 2350 Health Sciences Mall #1365, Vancouver, BC V6T 1Z3, Canada; Michael Smith Laboratories, University of British Columbia, 2185 East Mall, Vancouver, BC V6T1Z4, Canada; Michael Smith Laboratories, University of British Columbia, 2185 East Mall, Vancouver, BC V6T1Z4, Canada; Department of Microbiology and Molecular Genetics, University of California, Briggs Hall, 550 Storer Mall, Davis, CA 95616, United States; Michael Smith Laboratories, University of British Columbia, 2185 East Mall, Vancouver, BC V6T1Z4, Canada; Department of Microbiology & Immunology, University of British Columbia, 2350 Health Sciences Mall #1365, Vancouver, BC V6T 1Z3, Canada; Michael Smith Laboratories, University of British Columbia, 2185 East Mall, Vancouver, BC V6T1Z4, Canada; Department of Microbiology & Immunology, University of British Columbia, 2350 Health Sciences Mall #1365, Vancouver, BC V6T 1Z3, Canada; Michael Smith Laboratories, University of British Columbia, 2185 East Mall, Vancouver, BC V6T1Z4, Canada; Department of Biochemistry and Molecular Biology, University of British Columbia, Life Sciences Centre, 2350 Health Sciences Mall, Vancouver, BC V6T 1Z3, Canada

**Keywords:** host–microbe interactions, early-life microbiome, breastmilk IgA, microbiome development

## Abstract

Secretory Immunoglobulin A (SIgA) is the dominant mucosal antibody and a key regulator of the gut microbiota. In early life, infants rely on breastmilk as their primary source of SIgA, but the role of milk-derived SIgA in early life microbiota colonization dynamics remains incompletely understood. Here, we show that species-specific SIgA in milk is antigen-inducible and discriminates between closely related but immunologically diverging microbes in the neonatal gut. More specifically, milk species-specific SIgA promotes colonization by an anti-inflammatory *Escherichia coli* strain while restricting the expansion of pro-inflammatory *Proteus mirabilis*. These findings uncover a dual role of maternal milk SIgA in actively sculpting the early life gut microbiota with species-level precision.

## Introduction

The gut microbiota is highly dynamic during the early life period and plays a crucial role in immune development [1]. Feeding mode (breastfeeding vs. formula-feeding) is the primary determinant of microbiota composition during this window [2, 3]. Breastfeeding confers long-term health benefits, likely through shaping the infant microbiota [4, 5], but breastmilk is a complex mixture of many immune and nutritive components that may contribute to this phenomenon. Secretory IgA (SIgA), the most abundant antibody in the gut and an important regulator of gut microbiota composition [6], is also the most abundant immune component in breastmilk. The SIgA in breastmilk is produced by gut-primed plasma cells, and is thought to target the maternal gut microbiota [7]. We and others have shown that milk-derived SIgA can limit both pathogens and potential pathobionts previously encountered by the mother [8, 9]. Its influence over the dynamics of commensal and beneficial microbes in the offspring gut has not been fully elucidated.

In the present study, we used the *Pigr−/−* mouse model of SIgA deficiency, which does not secrete SIgA into the gut or milk [8, 10], to determine (i) whether milk SIgA reactivity can be reshaped through introduction of microbes and (ii) how bacteria-specific milk SIgA influences microbial colonization in the offspring gut.

## Materials and methods

### Animal husbandry and oral gavage

Heterozygous *Pigr+/−* micewereto obtain all mice. For *Enterobacteriaceae* colonization, female mice were orally gavaged once with 10^9^ Colony forming unit (CFU) of either *Escherichia coli* Mt1b1 or *Proteus mirabilis* in 200 μL of sterile PBS, both of which were isolated from healthy mice previously in our lab, at 4 weeks of age. Mice were checked for colonization by plating feces on MacConkey agar plates at 7 days and 14 days post-gavage. Stably colonized mice were bred at 6 weeks of age with non-colonized males. For postnatal colonization, pups were orally gavaged with 10^9^ CFU of either bacterial strain once every other day from Day 14 to Day 21 of life. Pups were sacrificed on Day 21 or Day 24 of life after weaning.

For cross-foster experiments, pups were removed from cages and placed in a holding container with bedding from the foster dam to transfer new dam scent. Pups were then placed in foster cages with dams and checked daily for 3 days to ensure successful foster and feeding in accordance with animal protocol #A21–0286.

### Milk collection

Milk was collected from dams at 12–14 days post-gestation. Mice were separated from their pups for 2 hours before collection. 80 mg/kg ketamine and 5 mg/kg xylazine administered by intraperitoneal injection was used to anesthetize mice. Once asleep, mice were administered 2 IU/kg Oxyto-Sure (Vetoquinol) to induce milk production. Mouse abdomens were cleaned with 70% isopropanol and milk was collected by manually pressing on teats and using a pipette to collect the secreted milk.

### Total SIgA ELISA

Total SIgA was measured in feces and milk as described previously [8].

### Species-specific SIgA ELISA

Species-specific SIgA ELISA was carried out as described previously [8] with the following modifications. *P. mirabilis* and *E. coli* Mt1b1 grown in Lysogeny Broth (LB) were diluted to an OD of one before washing with PBS and were fixed with 2% PFA for 30 minutes. Bacterial cells were then resuspended in sodium acetate buffer (pH 5.5) and used to coat a high-binding ELISA plate (Greiner). Fecal supernatant was diluted 1: 50 and milk samples were diluted 1:10 before adding to the plate to detect species-specific SIgA.

### Colony forming unit counting

*Bacteria* were quantified in mouse feces and tissues using dilution plating on MacConkey Agar plates. Tissues were homogenized in sterile PBS by vigorous shaking with a metal bead before dilution. CFU counts were normalized to tissue weight.

### Species-specific SIgA flow cytometry

Mouse fecal samples homogenized in PBS and centrifuged at 2000 g for 5 minutes before supernatant was collected and filter-sterilized to remove bacteria from SIgA in the sample. *E. coli* or *P. mirabilis* were grown in LB as described above to a concentration of 1e10^8^ CFU/mL. 1e10^6^ cells were then transferred to each well of a 96-well V-bottom plate. Fecal supernatant was then added to the bacteria and allowed to incubate for 30 minutes at room temperature to allow for SIgA binding. Cells were then washed twice with PBS + 1% BSA and resuspended in PBS + 1% BSA with PE labelled anti-mouse IgA diluted 1:40 to stain IgA and SYTO BC diluted 1:1000 to stain bacteria, and were incubated for 20 minutes on ice in the dark. Cells were then washed twice before analysis using a CytoFLEX L Analyzer. Compensation and visualization of results were done using FlowJo (version 10.10.1).

### CMT-93 cell infection

CMT-93 cells (mouse colonic epithelial cells) (ATCC CCL-223) were grown in DMEM+++ (DMEM, 10% v/v heat-inactivated fetal bovine serum (FBS), 1% v/v non-essential amino acids, and 1% v/v Glutamax) in T-75 flasks at 37°C in 5% CO_2_. Cells were split using 0.25% Trypsin when they reached 80–90% confluency (every 3–4 days) at a 1:6–1:10 dilution. Between passages 2 and 10, cells were seeded in 24-well plates at 50,000 cells per well in DMEM+++. Cells were allowed to reach confluency for 2 days before infection. *P. mirabilis* or *E. coli Mt1b1* were grown overnight in LB and sub-cultured to mid-log phase for 2 hours. Bacteria were then washed with PBS and diluted in DMEM+++ for infection at MOI 100. Bacteria were added to wells in 300uL of DMEM and left for 4 hours at 37°C in 5% CO_2_. Supernatant was collected and Bacteria were removed by centrifugation before storage at −80°C for further analysis.

### LDH cytotoxicity assay

LDH was quantified in cell supernatant using the CytoTox 96 Non-Radioactive Cytotoxicity Assay (Promega). Samples were not diluted. Vehicle and supernatant from uninfected cells were used as negative controls. Percent cytotoxicity was quantified using a positive control in which cells were all lysed using the provided lysis buffer to determine the maximum LDH output.

### Cytometric bead assay

Cytokines were quantified in cell supernatant using the Cytometric Bead Array (CBA) Mouse Inflammation Kit (BD Biosciences). Samples were not diluted. Samples were analyzed using the Attune Analyzer (Thermo Fisher). Results were obtained using FlowJo (version 10.10.1) with the CBA plugin.

### Immunohistochemistry

50 000 cells were seeded on 8-well chamber slides (ibidi, Cat. No. 80841) and infected under the conditions described above. After infection, cells were washed with PBS, fixed with 4% PFA, permeabilized with 0.1% Triton X-100, and blocked with 5% goat serum +1% BSA for 1 h at room temperature. Cells were incubated with primary antibodies (anti-ZO-1, Abcam, Cat. No. ab27613; anti-occludin, Zymed, Cat. No. 33–1500) for 2 h and secondary antibodies (Alexa Fluor 647 goat anti-rabbit, Cat. No. A-21245; Alexa Fluor 568 goat anti-mouse, Cat. No. A-11004; Thermo Fisher Scientific) for 50 min. Phalloidin (Thermo Fisher Scientific, Cat. No. A12379) and DAPI (Thermo Fisher Scientific, Cat. No. D21490) were used for actin and nuclear staining, respectively.

## Main

**Figure 1:**
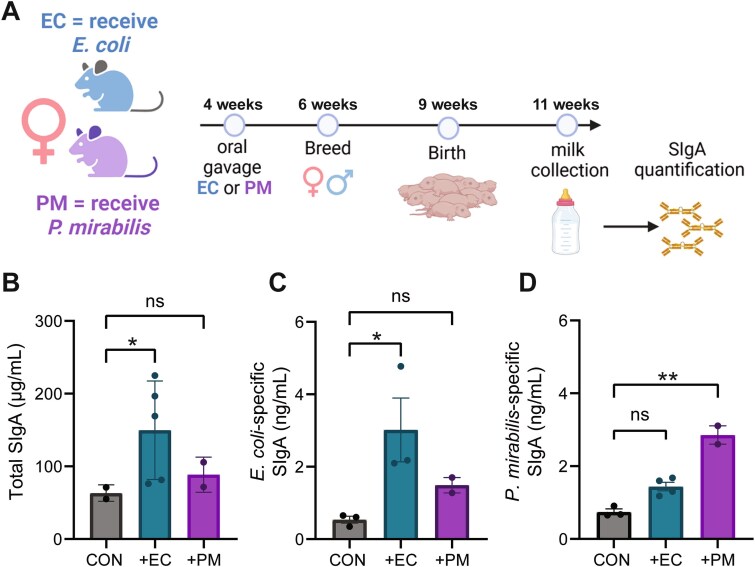
Induction of a species-specific SIgA response in milk through oral bacterial exposure. (A) Oral gavage and breeding timeline of female *Pigr+/−* mice. Mice were gavaged with *E. Coli* (EC) or *P. Mirabilis* (PM) at 4 weeks of age and bred at 6 weeks of age. Milk was collected from dams at 11 weeks of age, when pups were weaned, for SIgA quantification. (B) Total SIgA levels in milk of dams gavaged with *E. Coli* (EC), *P. Mirabilis* (PM), or neither (CON) quantified by ELISA. *P =* .0260 for comparison of EC and CON groups. *P =* .273 for comparison of PM and CON groups. (C) *E. Coli*-binding SIgA in milk of dams gavaged with EC, PM, or neither (CON) quantified by bacteria-specific ELISA. (D) *P. Mirabilis*-binding SIgA in milk of dams gavaged with EC, PM, or neither (CON) quantified by bacteria-specific ELISA. *P* values were determined by Kruskall–Wallis test with post-hoc Dunn’s test, which was chosen due to low sample size in the PM group, ^*^P < .05, ^**^P < .01, ns = not significant. *n* = 3 (CON), 4 (EC), or 2 (PM) dams. In (C), *n* = 3 (EC) due to limited milk quantity collected.

*Enterobacteriaceae* species are known to be the first colonizers of both humans and mice [11, 12], and are naturally absent in the mouse colony used in our study. To test whether gut microbial exposure alters the milk SIgA repertoire, we colonized *Pigr^+^/^−^* (SIgA^+^) female mice with *E. coli* Mt1b1, a proposed symbiont, or *P. mirabilis*, known to have pathobiont characteristics [13], prior to breeding (Fig. 1A). Both strains were isolated previously from stably colonized conventional mice [14]. Control mice received PBS and remained *Enterobacteriaceae*-free. Two weeks postpartum, milk from *E. coli*–colonized (EC) dams displayed higher total SIgA than milk from *P. mirabilis*–colonized (PM) or control dams *(P* = .0260), suggesting that *E. coli* induced a stronger overall milk SIgA response than *P. mirabilis* (Fig. 1B). This may be due to more immunogenic antigens present on *E. coli*, an enhanced ability of *E. coli* to interact with host cells compared to *P. mirabilis*, or other factors, which were not explored within this study. SIgA reactivity mirrored exposure: EC dams produced significantly more *E. coli*–specific SIgA (Fig. 1C). PM dams produced significantly more *P. mirabilis*–specific SIgA (Fig. 1D) in milk. When considered alongside total SIgA quantification, which showed that EC but not PM dams displayed elevated total milk SIgA, these suggest a numeric expansion of EC-specific SIgA in EC dams, driving an elevated total SIgA titer, and a shift in SIgA specificity in PM dams, leading to elevated PM-specific SIgA without an increase overall SIgA titer. In both cases, dams gavaged with the alternative *Enterobacteriaceae* species also displayed numerically higher species-binding SIgA (PM-gavaged dams displayed higher EC-specific SIgA than control dams), although differences were not significant. This suggests an overall heightened anti-*Enterobacteriaceae* SIgA response in dams gavaged with either species, and may be due to shared epitope structure between the two species, or cross-reactivity of SIgA. The use of ELISA to capture species-specific reactivity limits our ability to define targeted epitopes and prevents us from confirming the quantity of species- versus family-specific SIgA. The low sample size in the PM group is also a limitation that prevents deeper comparison of SIgA induction between bacterial species. However, these findings do show that maternal microbial exposure prior to pregnancy drives the production of significantly heightened species-specific SIgA in milk, with potential consequences for vertical microbial transmission.

To confirm that milk is the primary source of early life intestinal SIgA, we measured fecal SIgA in *Pigr^+^/^−^* (SIgA^+^) pups born to either SIgA^+^ or SIgA^−^ dams, before (Day 19) and after (Day 24) weaning. Pups nursed by SIgA^+^ dams had higher SIgA levels before, but not after, weaning, illustrating that milk is the dominant source of intestinal SIgA in early life (Fig. S1A–B). After weaning, pups began to produce their own SIgA and were no longer reliant on maternal SIgA (Fig. S1B). In fact, SIgA+ pups fed by SIgA- dams display an elevated fecal SIgA response in shortly after weaning, supporting a premature activation of the gut SIgA compartment in the absence of maternal antibodies in early life. This is consistent with findings in formula-fed infants, who exhibit elevated fecal SIgA levels compared to breastfed infants [8, 15], and may be due to altered microbiota composition or increased contact between intestinal microbes and the host epithelium in the absence of maternal SIgA.

Although milk SIgA is known to generally limit *Enterobacteriaceae* overgrowth in infants and neonatal mice, prior studies have lacked species- or strain-level resolution in assessing *Enterobacteriaceae*-SIgA relationships [9, 16]. Although this family can contribute to disease and inflammation in some contexts [9, 13], *Enterobacteriaceae* can also promote protective responses in the gut [17, 18], illustrating their functional diversity. *Enterobacteriaceae* are also consistently among the first colonizers of the neonatal gut in both human infants and mice [11], hinting at their relevance in early microbial succession and host development.

**Figure 2:**
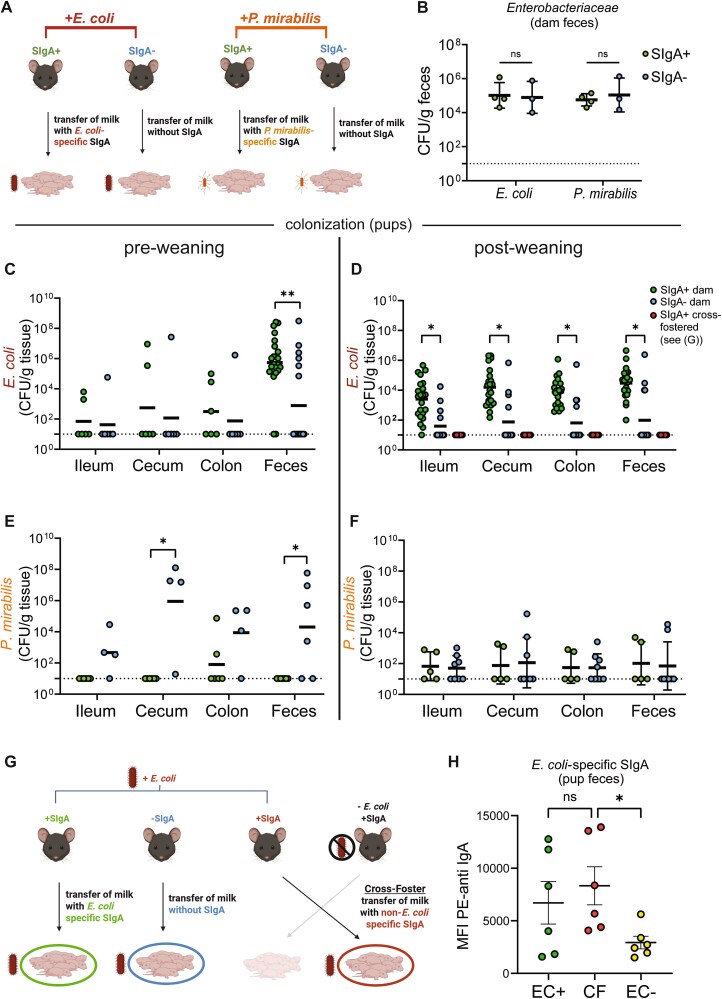
Milk SIgA promotes *E. Coli* transfer but limits *P. Mirabalis* transfer from dam to offspring. (A) Experimental design. SIgA+ or SIgA- dams were gavaged with either *E. Coli* or *P. Mirabilis* 2 weeks prior to breeding. This led to 4 groups of pups. Pups received milk with or without SIgA, and were born to dams colonized with either *E. Coli* or *P. Mirabilis.* (B) *E. Coli* or *P. Mirabilis* quantified by CFU plating in the feces of dams after oral gavage and breeding. SIgA+ dams are compared to SIgA- dams orally gavaged with the same bacterial speices (either *E. Coli* or *P. Mirabilis*). Colonization levels are similar between SIgA+ and SIgA- dams, and between dams gavaged with either species. (C–D) *E. Coli* quantified by CFU counting in the feces and intestinal tissues of pups born to SIgA+ or SIgA- *E. Coli* colonized dams at 19 days (C) or 24 days (D) of age. (D) Also includes a cross-fostered group of pups (see [G]). (E–F) *P. Mirabilis* quantified by CFU counting in feces and intestinal tissues of pups born to SIgA+ or SIgA- *P. Mirabilis* colonized dams at 19 days (E) or 24 days (F) of age. (G) Cross-foster experimental design for the generation of the red-labelled group in Fig. 2D. SIgA+ or SIgA- dams were gavaged with *E. Coli* or PBS (− *E. Coli*) before breeding. At 5 days of age, pups born to an SIgA+ *E. Coli* + dam were cross-fostered to an *E. Coli-* SIgA+ dam. Cross-fostered pups would obtain *E. Coli* throught the birthing process, but would receive milk lacking SIgA for the majority of the milk-feeding period. (H) Quantification of *E. Coli-*specific SIgA in pup feces at 4 weeks of age, measured by flow cytometry. MFI = mean fluorescence intensity of PE anti-IgA staining of *E. Coli* cells.

After establishing that maternal exposure to either *E. coli* or *P. mirabilis* induces species-specific milk SIgA, we next examined how this antibody response shapes microbial transmission to offspring (Fig. 2A). Dams colonized with either strain showed similar bacterial loads regardless of SIgA status, indicating that gut SIgA production does not affect maternal colonization (Fig. 2B). However, milk SIgA had divergent effects on vertical transmission. Unexpectedly, pups born to EC SIgA^+^ dams (*Pigr+/−* dams gavaged with *E. coli* before breeding), who would receive *E. coli-*specific SIgA through milk and *E. coli* through vertical transmission, had higher *E. coli* levels, both before and after weaning, compared to pups from EC SIgA^−^ dams (Fig. 2C–D). In fact, most pups nursed by EC SIgA^−^ dams failed to acquire *E. coli* at all, revealing that milk SIgA actively promotes transfer of specific symbionts from mother to offspring.

In direct contrast to *E. coli*, we observed a negative relationship between milk SIgA and *P. mirabilis*. Pups born to PM SIgA^−^ dams exhibited a bloom of *P. mirabilis* before weaning, which was lost after weaning (Fig. 2E–F). This aligns with prior studies showing that SIgA restricts *Enterobacteriaceae* overgrowth, and supports a key role for milk SIgA in limiting pathobiont expansion.

To determine whether these opposing effects were due to bacteria-specific SIgA, rather than total SIgA presence, we cross-fostered pups born to EC SIgA^+^ dams to SIgA^+^ control dams, which lack *E. coli* or *E. coli-*specific SIgA*,* at Day 5 (Fig. 2G). These pups were briefly exposed to *E. coli* from their birth dam but received SIgA+ milk that lacked *E. coli-*specific SIgA during the remainder of the nursing period. By Day 24, cross-fostered pups mounted elevated *E. coli*–specific SIgA responses, confirming early exposure to *E. coli* (Fig. 2H). However, none of the cross-fostered pups retained *E. coli* colonization, mirroring pups nursed by EC SIgA^−^ dams. Thus, the presence of SIgA alone is insufficient to limit *E. coli*. Instead, *E. coli* colonization requires maternal *E. coli* carriage and ongoing delivery of *E. coli*–specific SIgA via milk. These findings reveal that species-specific milk SIgA can actively promote colonization of select commensals in the neonatal gut.

Milk-derived SIgA had opposing effects on two closely related species, promoting commensal *E. coli* and restricting *P. mirabilis*, despite both belonging to the same phylogenetic family. This divergence was observed within the same mouse model. The cytokine milieu induced by a microbe plays an important role in determining the quantity, affinity, and effects of the induced SIgA [19, 20]. To determine whether these two bacterial species set off different epithelial immune programs, we infected cultured mouse intestinal epithelial cells with either species. *P. mirabilis* induced cytotoxicity and elevated IFN-γ, a pro-inflammatory cytokine, consistent with its pathobiont status and inflammatory capacity. In contrast, *E. coli* triggered IL-10 production and was non-cytotoxic, supporting an immunoregulatory effect and classification as a well-tolerated or even symbiotic commensal (Fig. S2A–C). *P. mirabilis* also drove more severe epithelial cell damage, including disruption of tight junction structure, compared to *E. coli,* which induced actin reorganization but no loss of tight junction structure (Fig. S2D)*.* Although additional work is needed to fully map the microbial cues driving SIgA responses, these data suggest that divergent epithelial immune signaling may underlie the opposing SIgA-microbe dynamics observed between *E. coli* and *P. mirabilis*, and supports the capacity of SIgA to discriminate between pro- and anti-inflammatory microbes.

This study reveals a fundamental, dual role for milk-derived SIgA: it can both promote symbionts and restrict inflammatory microbes within the early life gut. These opposing effects were observed across closely related *Enterobacteriaceae* species within the same mouse model, highlighting the nuanced, species-specific functions of milk SIgA and reflecting the functional diversity within this bacterial family. Our findings demonstrate that maternal milk SIgA is a powerful and active regulator of *Enterobacteriaceae* community structure in the neonatal gut, shaping its composition in context-dependent and distinct ways.

Beyond defining a key mechanism of maternal–infant microbiota crosstalk, this work establishes a robust model for better understanding milk SIgA’s function in the early life gut. Future efforts to identify microbes that are selectively enhanced or excluded by SIgA could pave the way for maternal probiotic strategies designed to work in synergy with this naturally occurring component of breastmilk. Harnessing the capacity of milk SIgA to facilitate or control microbial transfer from mother to infant, interventions may be developed to promote immune maturation and support the long-term health of infants via breastmilk SIgA.

Together, this study positions milk SIgA as a critical mediator of early life microbial assembly, with far-reaching implications for maternal–infant health and microbiota-targeted interventions.

## Supplementary Material

Supplementary_Data_revisions-Nov25_wraf266

## Data Availability

All data generated or analysed during this study are included in this published article [and its supplementary information files].
