# Supplementary material for: Milk IgA promotes symbionts and limits pathobionts in the early life gut
Source: ISME J. 2025 Dec 1;19(1):wraf266. doi: 10.1093/ismejo/wraf266 (PMC12704429; doi:10.1093/ismejo/wraf266)
Supplement: Supplementary_Data_revisions-Nov25_wraf266 [file supplementary_data_revisions-nov25_wraf266.docx]

**Supplementary Figures:**


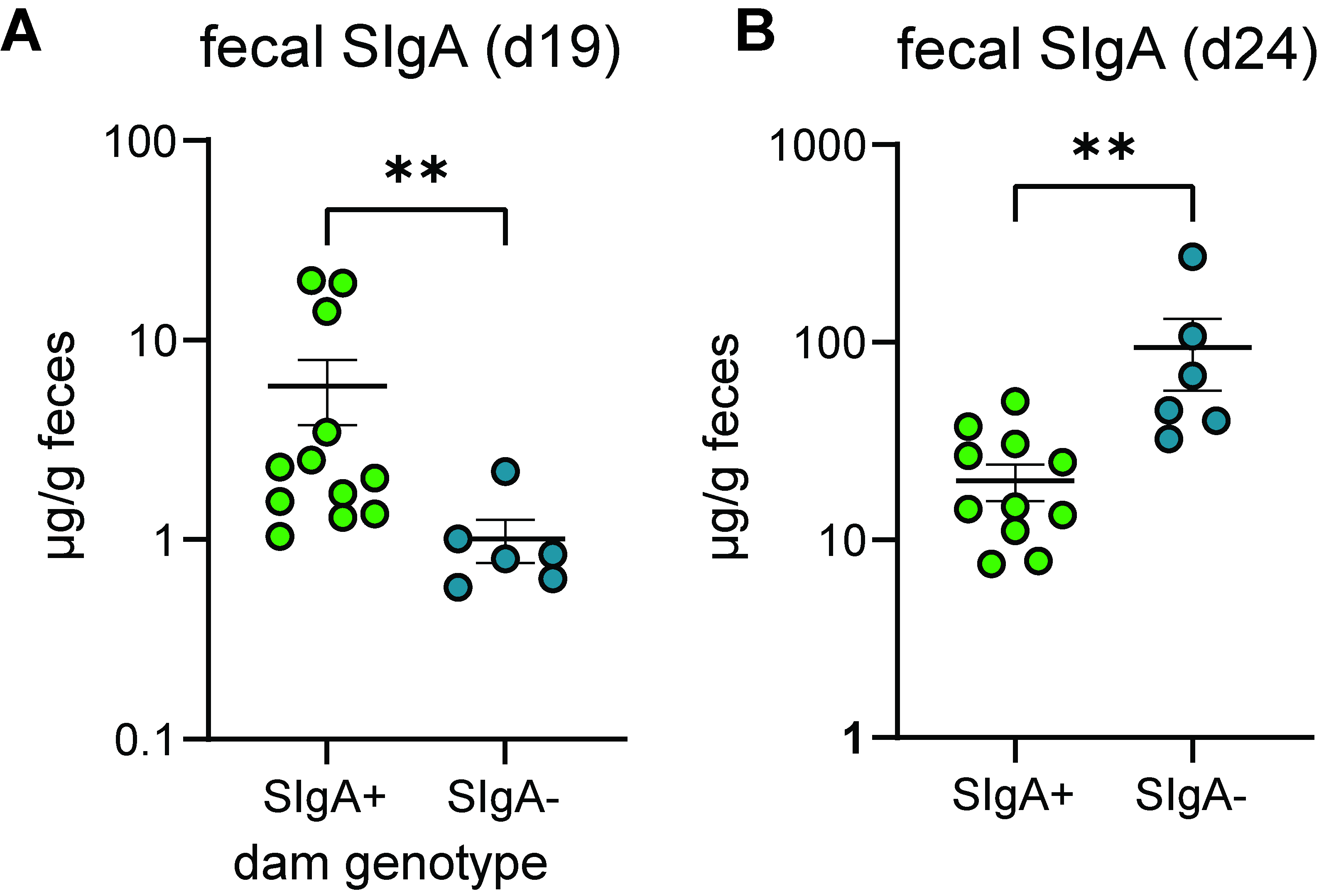


**Figure S1.** **SIgA is transferred from dam to offspring until weaning.**

(A) SIgA in feces of pups born to SIgA+ or SIgA- dams at 19 days of age, 2 days before weaning.

(B) SIgA in feces of pups born to SIgA+ or SIgA- dams at 23 days of age, 2 days after weaning.

Statistical significance assessed by Mann-Whitney U-test. **p<0.01


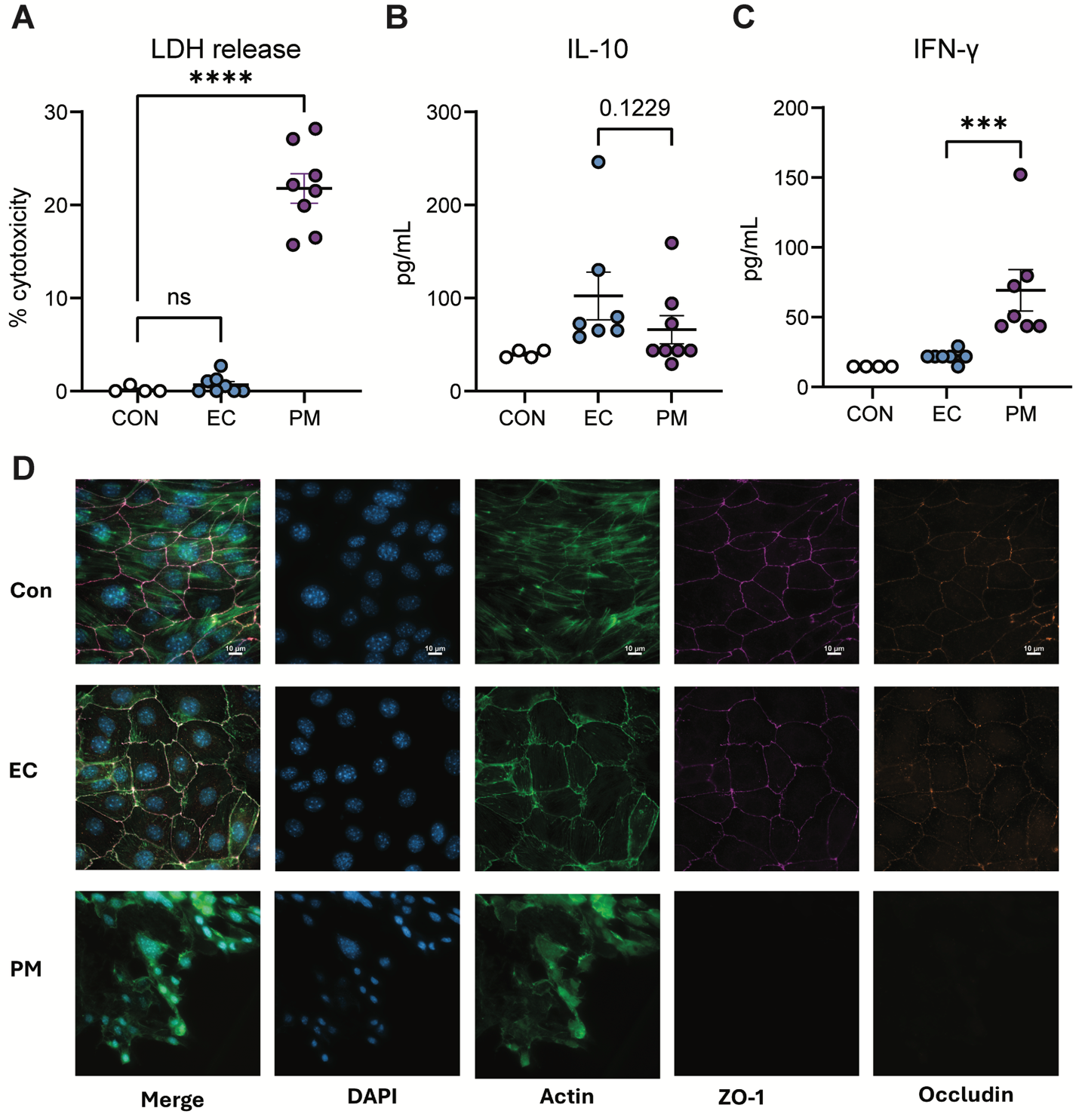


**Figure S2. *E. coli* and *P. mirabilis* induce divergent responses in the mouse gut epithelium**

(A) % cytotoxicity of *E. coli* (EC) or *P. mirabilis* (PM) in CMT-93 cells infected for 4 hours at MOI 100. CON = uninfected cells.

(B-C) IL-10 (B) and IFN-γ (C) measured by cytometric bead assay in supernatant from EC or PM infected CMT-93 cells as in (A).

(D) Immunohistochemistry of CMT-93 cells infected with EC or PM as in (A). Nuclei are stained using DAPI. Cells were also stained for visualization of actin, and the tight junction proteins ZO-1 and Occludin.

Statistical significance determined by Mann Whitney U-test to compare 2 groups.
